# Nano-Titanium Dioxide Induces Ovarian Function Damage in Mice by Mediating Granulosa Cell Apoptosis

**DOI:** 10.3390/ijms26146981

**Published:** 2025-07-20

**Authors:** Jie Chen, Yaxuan Zhang, Shengbo Zhang, Changbao Wu, Jingyu Ren, Xiaoxiao You, Yanfeng Dai

**Affiliations:** 1 School of Life Science, Inner Mongolia University, Hohhot 010000, China; chenjie@immu.edu.cn (J.C.); 18047159182@163.com (Y.Z.); 15034954790@163.com (S.Z.); neidashengke@sina.com (C.W.); renjingyu2014@163.com (J.R.); yxx200929@163.com (X.Y.); 2 College of Basic Medicine, Inner Mongolia Medical University, Hohhot 010000, China

**Keywords:** nano-TiO_2_, ovary, granulosa cell, apoptosis, ROS

## Abstract

The accumulation of nanoparticles (NPs) in the female body has raised global concerns regarding potential effects on the reproductive system. This study aimed to investigate the toxic effects of nano-titanium dioxide (nano-TiO_2_) exposure on the ovaries and the underlying mechanisms. By establishing a nano-TiO_2_ accumulation model in mice, our research systematically evaluated the effects of different concentrations of nano-TiO_2_ exposure on the development and reproductive endocrine functions of mice. The results showed that nano-TiO_2_ exposure significantly reduced the littering rate, sex hormone levels, and ovarian index of mice, and the effects were dose-dependent. Studies on the mechanisms involved revealed that nano-TiO_2_ induces an excessive production of reactive oxygen species (ROS), leading to the potential collapse of the mitochondrial membrane and an increase in the apoptosis rate of granulosa cells, thereby triggering oxidative stress and inhibiting the expression of ovarian-specific genes and granulosa-cell function genes. This study reveals the “dual blow” mechanism of nano-TiO_2_-mediated ovarian morphology and function through oxidative stress in granulosa cells, namely directly disrupting cellular homeostasis and interfering with the reproductive-related gene network, ultimately leading to decreased ovarian function. This provides experimental evidence for assessing the reproductive risks of nanomaterials in women.

## 1. Introduction

Due to the small particle diameter and particular structure of nanomaterials, as well as their novel physical and chemical properties, the application of nanomaterial technology has become a symbol of 21st-century science and technology, with nanoscience regarded as one of the three pillars of science [1]. Titanium dioxide is the natural oxide of metallic titanium, with a diameter of less than 100 nm. It not only has low toxicity and mild biological effects but is also classified as a bioinert material. As a result, it is widely used in clinical treatment [2,3]. Due to its potential advantages in new medical treatments, it has also become an increasingly important medical material [4]. Furthermore, nano-TiO_2_ is widely used in various aspects of daily life [5], such as purifying air, treating wastewater, processing food, making toothpaste and sunscreen, etc. [6]. It can enter the human body via the food chain and other means, as well as through the respiratory tract, digestive tract, etc. [7,8,9]. Therefore, much attention has been paid to the potential hazards of nano-TiO_2_ in the body. Findings from epidemiological studies suggest that following chronic low-dose human exposure, nano-TiO_2_ may enter the systemic circulation, cross the blood–brain barrier, and accumulate in diverse tissues and organs, potentially leading to adverse health effects [10,11], which aligns with longstanding scientific concerns regarding its potential health implications. After mice consume nano-TiO_2_ over a long period of time, it can pass through the ovaries and be deposited in the follicles, changing the structure of the ovarian tissue, reducing the number of oocytes, inducing ovarian cysts, causing disorder in follicle development, and resulting in a decrease in the fertilization rate of oocytes, severely affecting embryo development [12].

Nano-TiO_2_ triggers the cellular oxidative stress response, causing the cell-membrane lipid layer to rupture due to oxidative stress [13], resulting in DNA oxidative damage within the cells and the formation of micronuclei [14]. When 20–35 nm diameter nano-titanium dioxide particles enter human keratinocytes, cytoplasmic dissolution occurs, and mitochondrial structures become disordered and swell and disintegrate; additionally, the nuclear periplasmic space expands, and the nucleus shrinks, indicating that the nano-TiO_2_ particles can damage the ultrastructure of the cells and inhibit cell growth [15].

Apoptosis, as a programmed cell death mechanism, can actively eliminate damaged cells and is an important mechanism for the body to complete tissue repair and self-renewal [16]. When nanoparticles within cells in an aqueous medium are irradiated with ultraviolet light, a series of ROS are produced, thereby inducing cell apoptosis [17,18]. That is, nanoparticles can induce cell apoptosis in a special way, affect the programmed cell death mechanism, and cause damage to organs and tissues. It is reported that the mitochondrial pathway is the main pathway of cell apoptosis. Excessive ROS can induce the occurrence of oxidative stress response and promote the initiation of apoptosis [19,20,21,22]. The occurrence of ROS within the cells, along with the apoptosis of certain granular cells, indicates that ROS are closely related to the apoptosis of granular cells [23]. The above pathway may be important in helping nanoparticles to induce the apoptosis of ovarian cells and damage the structure and function of follicles.

It has been confirmed that nano-TiO_2_ can deposit in the body through specific pathways. However, at present, no in-depth research or reports on the biological effects and cellular response mechanisms of nano-TiO_2_ have been published. The recovery of ovarian function after the occurrence of damage caused by the deposition of nano-TiO_2_ in the body remains an important issue to be addressed in the field of ecological safety and environmental toxicology. Given the potential harm that the deposition of nano-TiO_2_ in the body may pose to the female reproductive system, this study uses a mouse accumulation model to conduct research on aspects such as the hormone secretion level of the damaged ovary, morphological structure changes, littering rate, and apoptosis of granulosa cells. Our findings demonstrate that different concentrations of nano-TiO_2_ have varying effects on the reproductive performance of mice, providing a scientific basis for determining the safe dosage of nanomaterials for human use and providing a theoretical basis and laboratory research evidence for subsequent studies and the clinical treatment of potential female reproductive system diseases caused by nano-TiO_2_.

## 2. Results

### 2.1. The Effects of Nano-TiO_2_ on the Growth and Birth Rate in Mice

**Figure 1:**
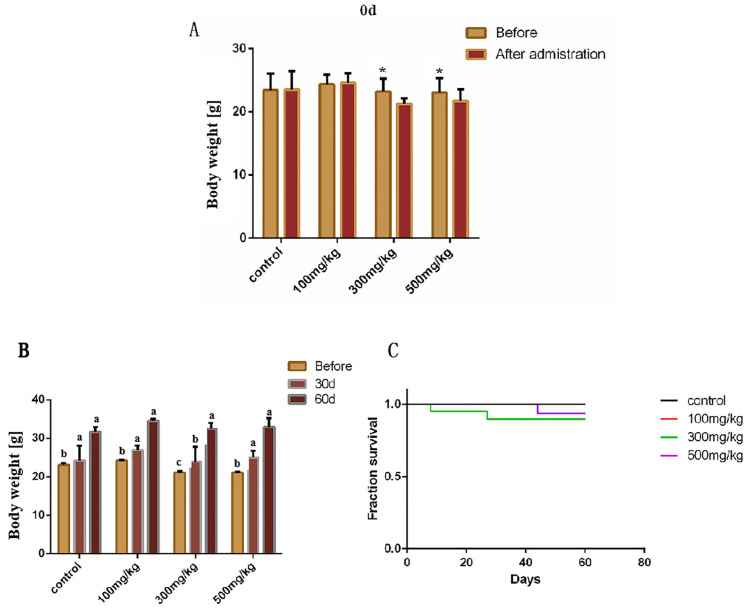
The effects of in vivo accumulation of nano-TiO_2_ on the growth of mice (weight unit: g). (**A**). The results obtained by weighing the mice in each group before infusion and after continuous infusion for 10 days. * indicates significant difference (*p* < 0.05, *n* = 5). (**B**). The results obtained by weighing the mice in each group before infusion and 30 and 60 days after infusion. a, b, c indicates significant difference (*p* < 0.05, *n* = 5). (**C**). The survival rates of the mice in each group were continuously observed for 60 days after infusion with nano-TiO_2_, (*p* < 0.05, *n* = 5).

The weight changes in mice in each group before and after infusion of nano-TiO_2_ were statistically analyzed. The results showed that the weight of mice in the 300 mg/kg group and the 500 mg/kg group decreased significantly before and after infusion (*p* = 0.024) (Figure 1A). At the 30th and 60th days after drug withdrawal, the weight changes in mice in each group were not significant (Figure 1B). The survival curves were plotted by recording the number and time of mouse deaths throughout the experiment. The results showed that the higher the nano-TiO_2_ concentration, the lower the mortality rate (Figure 1C).

**Figure 2:**
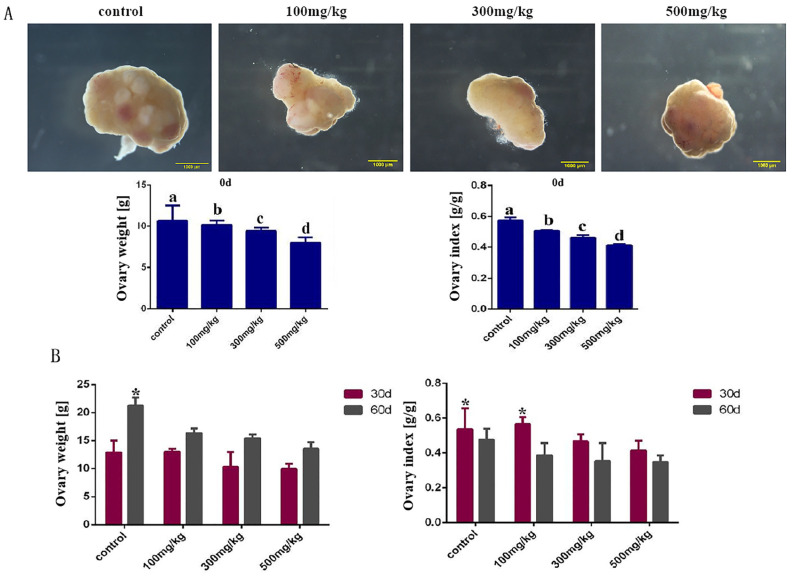
Changes in the ovarian weight and ovarian index in each group of mice after infusions of different concentrations of nano-TiO_2_ (1000 μm) (weight unit: g). (**A**). The ovarian weight and ovarian index of mice in each group after a continuous infusion of nano-TiO_2_ for 10 days. In the bar chart, a, b, c, and d represent comparisons between the two groups, and the differences are significant (*p* < 0.05, *n* = 5). (**B**). The ovarian weight and ovarian index of mice in each group 30 and 60 days after infusion were continuously observed. In the bar chart, * indicates a significant difference when compared with other groups (*p* < 0.05, *n* = 5).

We collected the ovaries of the mice after administration and weighed them, calculating the ovarian index (ovarian index = ovarian weight/body weight). The results showed that the ovarian index of the mice in the 100 mg/kg infusion group decreased significantly (*p* = 0.017) when compared with the control group, and the ovarian index reached the minimum value when the infusion concentration was 500 mg/kg (*p* = 0.001). The infusion concentration was inversely proportional to the ovarian index, and nano-TiO_2_ inhibited ovarian development (Figure 2A). By measuring and calculating the ovarian weight and index of the ovaries on the 30th and 60th days after infusion, the results showed that the ovarian weight of the experimental group was significantly lower than that of the control group (*p* < 0.05), and the ovarian index still showed a downward trend (Figure 2B).

**Figure 3:**
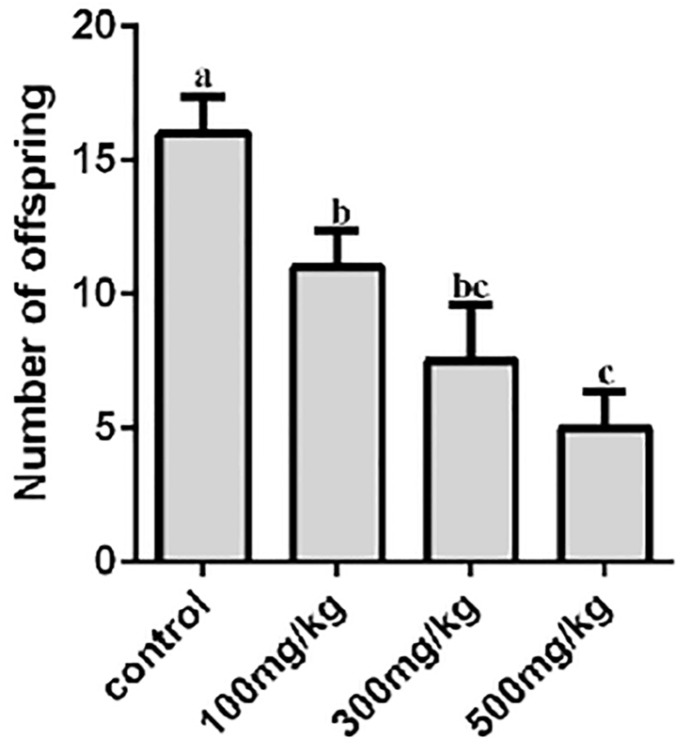
The number of pups born per litter in each group of mice after being infusion with different concentrations of nano-TiO_2_ in the stomach. In the column chart, a, b, and c represent significant differences in data between the two groups (*p* < 0.05, *n* = 5).

The results of the birth rate showed that compared with the control group, the birth numbers of mice in the 100 mg/kg group (*p* = 0.037), 300 mg/kg group (*p* = 0.006), and 500 mg/kg group (*p* = 0.002) all significantly decreased (*p* < 0.05). The administration of nano-TiO_2_ significantly affected the reproductive ability of mice (Figure 3).

### 2.2. The Effects of Nano-TiO_2_ on the Reproductive Endocrine System of Mice

**Figure 4:**
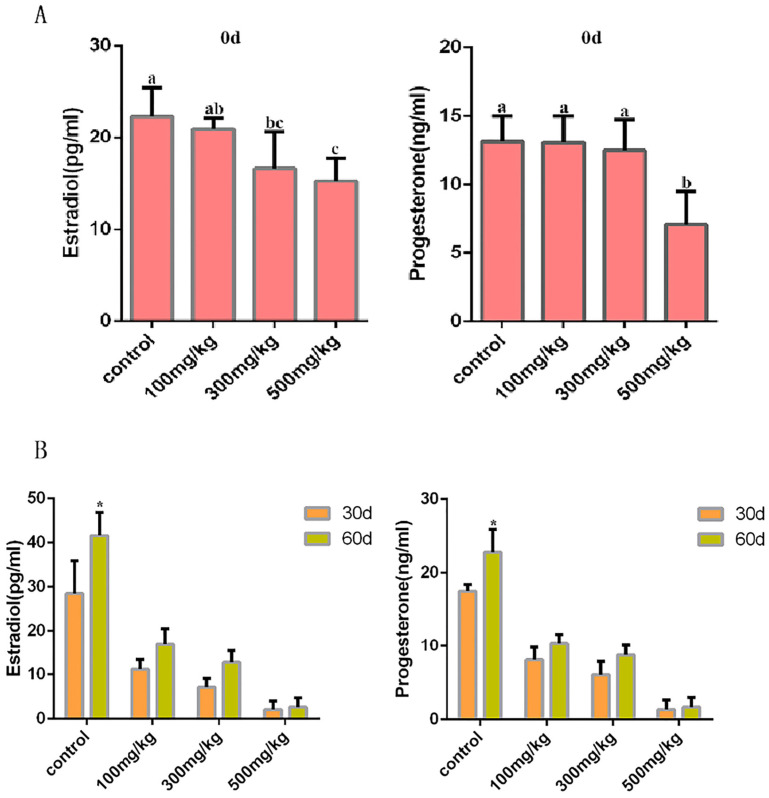
The effect of an in vivo accumulation of nano-TiO_2_ on the secretion of female hormones in mice. (**A**). The levels of serum estrogen and progesterone in each group of mice after infusion. In the column chart, a, b, and c represent significant differences in data between the two groups (*p* < 0.05, *n* = 5). (**B**). The levels of serum estrogen and progesterone in each group of mice at 30 days and 60 days after infusion. In the bar chart, * indicates a significant difference when compared with other groups, (*p* < 0.05, *n* = 5).

To investigate whether nano-TiO_2_ affects the synthesis and secretion of ovarian hormones, we measured the concentrations of estradiol and progesterone in the serum of each group of mice. The statistical results showed that compared with the control group, the concentrations of estradiol in the serum of mice in the 300 mg/kg group *(p* = 0.042) and the 500 mg/kg group (*p* = 0.018) were significantly lower (*p* < 0.05). Compared with the control group, the concentration of progesterone in the serum of mice in the 500 mg/kg group was significantly lower (*p* = 0.008) (Figure 4A). The concentrations of estradiol and progesterone in the serum of each group of mice 60 days after stopping the infusion of nano-TiO_2_ were higher than those 30 days after stopping the infusion, except for the 500 mg/kg group. After 30 days and 60 days without infusion, the concentrations of estradiol and progesterone in the serum of the experimental group were lower than those of the control group, *p* < 0.05. In the 500 mg/kg group, the estradiol and progesterone levels in the serum of mice after 30 days and 60 days without infusion showed no change (Figure 4B).

### 2.3. Histological Changes in Mouse Ovarian After Infusion of Nano-TiO_2_

**Figure 5:**
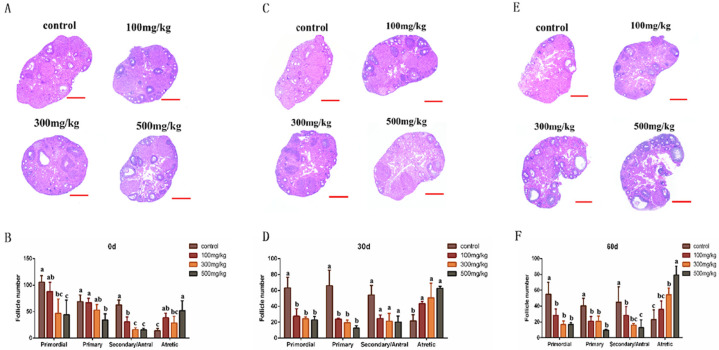
An observation of the histological changes in the mouse ovaries caused by the accumulation of nano-TiO_2_ in vivo (500 μm). (**A**). The results of HE staining of the ovaries of mice in each group after a continuous infusion of nano-TiO_2_. (**B**). The total follicles at all levels of the ovaries of mice in each group after infusion. (**C**). Histological changes in the ovarian tissues of mice in each group 30 days after infusion. (**D**). The statistical results of the number of follicles at all levels of the ovaries in each group 30 days after infusion. (**E**). Histological changes in the ovarian tissues of mice in each group 60 days after infusion. (**F**). The total number of different types of follicles in each group 60 days after infusion. In the bar chart, a, b, c, and represent significant differences in data between the two groups (*p* < 0.05, *n* = 5).

After continuous infusion for 10 days, the ovarian tissues of mice in each group were collected for histological observation and follicle counting. Compared with the control group, the ovarian diameters in the experimental groups were smaller, the ovarian tissue structure was loose, and the arrangement of granulosa cells was disordered. The statistics of the number of follicles at all levels showed that, compared with control group, the number of primordial follicles in 300 mg/kg group (*p* = 0.011) and the 500 mg/kg group (*p* = 0.009) was significantly reduced (*p* < 0.05); the number of primary follicles was significantly reduced in the 500 mg/kg group (*p* = 0.005); the number of secondary/mature follicles was significantly reduced in the 100 mg/kg group (*p* = 0.008), the 300 mg/kg group (*p* = 0.003), and the 500 mg/kg group (*p* = 0.001) (*p* < 0.01); and the number of atretic follicles was significantly increased in the 100 mg/kg group (*p* = 0.041) and the 500 mg/kg group (*p* = 0.005) (Figure 5A,B).

Thirty days after infusion, histological changes in the ovarian tissues in each group were observed. It was found that with the increase in infusion dose, the number of follicles in the medulla of the ovary decreased significantly, the number of interstitial cells increased significantly, and the medulla structure became loose and vacuolated (Figure 5C). The results of follicle count showed that compared with the control group, the number of follicles at all levels in the experimental group decreased, and the number of closed follicles increased (*p* < 0.05) (Figure 5D). Sixty days after the infusion, the vacuolation in the medulla of the ovary was more obvious (Figure 5E), and the results of the follicle count were consistent with the trend at 30 days after the infusion (Figure 5F). That is, the damage to the ovary caused by nano-TiO_2_ could not be effectively recovered over time.

### 2.4. An Analysis of mRAN Levels in Ovarian Tissue After Nano-TiO_2_ Infusion and the Detection of Apoptotic Execution Proteins and Their Localization

We examined the expression levels of two classes of reproductive-specific genes in the ovary, namely those expressed specifically in oocytes and those expressed specifically in granulosa cells. The number of oocyte-specific expressed genes (*Oct4*, *Bmp15*, *Gdf9*, *Foxo3*, *Zp3*) and granulosa-cell-specific expression genes (*Foxl2*, *Inhibinα*) were determined in order to evaluate the effect of nano-TiO_2_ on ovarian oocytes and granulosa cells. At the same time, the expression levels of apoptosis-specific genes (*Bax* and *Bcl-2*) were detected. In addition, the expression levels of antioxidant enzyme genes (*Sod2*, *Cat*, and *Gpx*) were used to analyze the degree of apoptosis in ovarian tissue and the degree of ovarian peroxidative damage after ovarian exposure to nano-TiO_2_. *GAPDH* was used as the reference gene.

**Figure 6:**
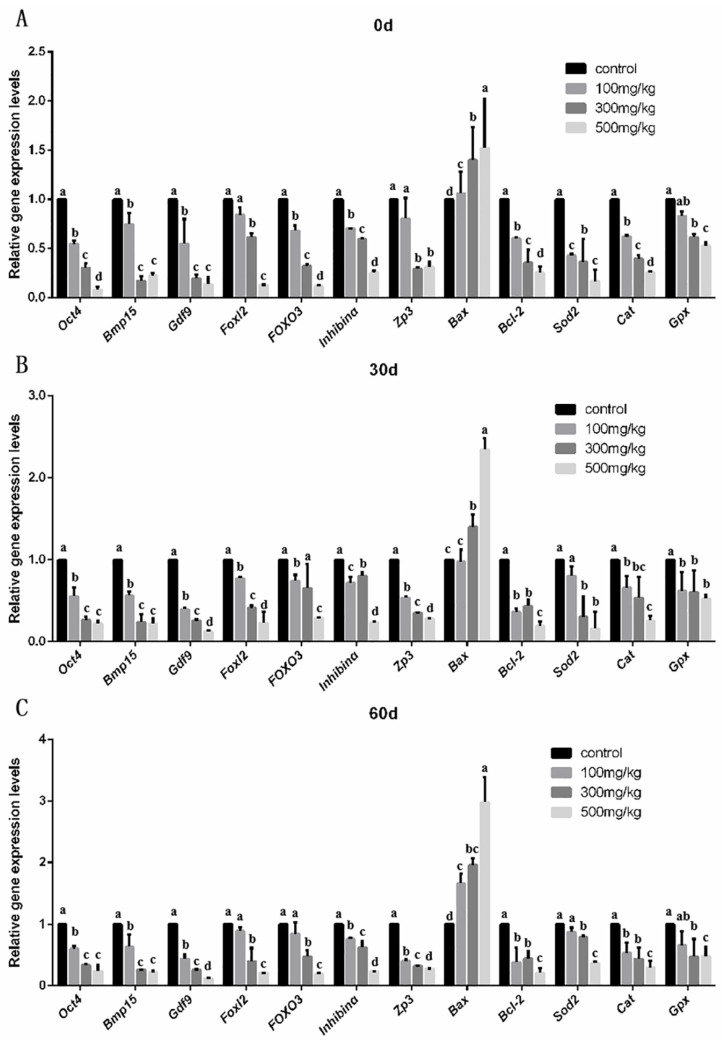
The mRNA levels in ovarian tissue after in vivo accumulation of nano-TiO_2_. (**A**). An analysis of specific gene expression in oocytes and granulosa cells after continuous infusion. (**B**). An analysis of specific gene-expression levels in the ovary at 30 days after infusion. (**C**). An analysis of specific gene-expression levels in the ovary at 60 days after infusion. In the bar charts, a, b, c, d represent significant differences between the two groups (*p* < 0.05, *n* = 5).

At 0 d, the mRNA expressions of the oocyte-specific genes *Oct4* (*p* = 0.001), *Bmp15* (*p* = 0.006), *Gdf9* (*p* = 0.001), and *Foxo3* (*p* = 0.001), the zona pellucida-specific gene *Zp3* (*p* = 0.001), and the granulosa cell-specific genes *Foxl2* (*p* = 0.008) and *inhibinα* (*p* = 0.009) were significantly lower than those in the control group (*p* < 0.05). For the apoptosis-related genes, as the concentration of nano-TiO_2_ increased, the expression level of the anti-apoptotic gene *Bcl-2* (*p* = 0.016) decreased, while the expression level of the pro-apoptotic gene *Bax* (*p* = 0.002) increased. The expression levels of the specific antioxidant enzyme genes *Sod2* (*p* = 0.009), *Cat* (*p* = 0.001), and *Gpx* (*p* = 0.001) all decreased in the cells, indicating that a severe oxidative stress response occurred in the ovary as the concentration of nano-TiO_2_ increased (Figure 6A). At 30 d and 60 d, these genes showed the same trend in expression as at 0 d, that is, the oxidative stress response in the ovary persisted after the infusion was stopped (Figure 6B,C).

**Figure 7:**
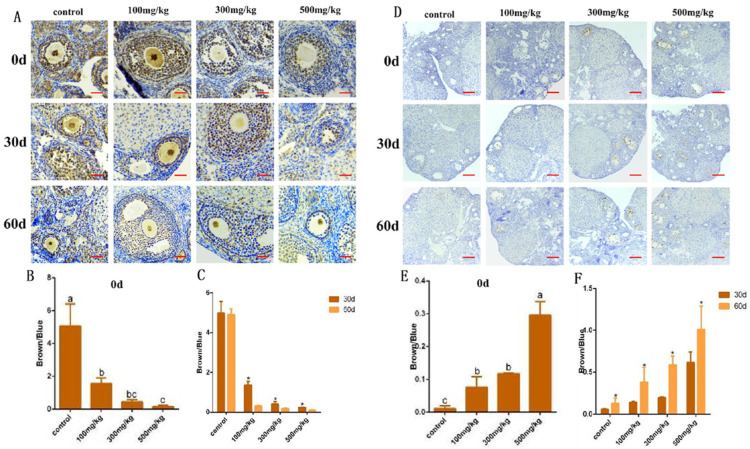
An analysis of the localization and expression of apoptosis-related proteins in ovarian tissue after nano-TiO_2_ deposition. (**A**–**C**). Changes in the localization and expression of PCNA protein in ovarian tissues of each group at different infusion times (The scale bar of (**A**) is 200 μm). (**D**–**F**). The localization and expression of caspase-3 in damaged ovarian tissues of each group at different times after infusion (the scale bar of (**D**) is 100 μm). In the bar charts (**B**,**E**), a, b, and c represent significant differences between the two groups (*p* < 0.05, *n* = 5). In the bar chart (**C**,**F**), * indicates a significant difference when compared with other groups, (*p* < 0.05, *n* = 5).

To identify apoptotic cells, we conducted immunohistochemical assays using PCNA and caspase-3. The results showed that at 0 days, the positive expression of PCNA in the experimental group was significantly lower than that in the control group (*p* = 0.001). As the infusion concentration increased, the positive expression of PCNA in the ovarian tissue gradually weakened (*p* < 0.05). At 30 days and 60 days, the number of PCNA-positive cells in the ovaries of the experimental group was significantly lower than that in the control group (*p* = 0.001) (Figure 7A–C). Regardless of the time point, whether at 0 days, 30 days, or 60 days, the positive expression of c-3 in the experimental group was significantly higher than that in the control group, and the positive expression of caspase-3 was proportional to the infusion concentration (*p* = 0.001) (Figure 7D–F).

### 2.5. Determining the Effect of Nano-TiO_2_ on Promoting the Apoptosis of Granulosa Cells

**Figure 8:**
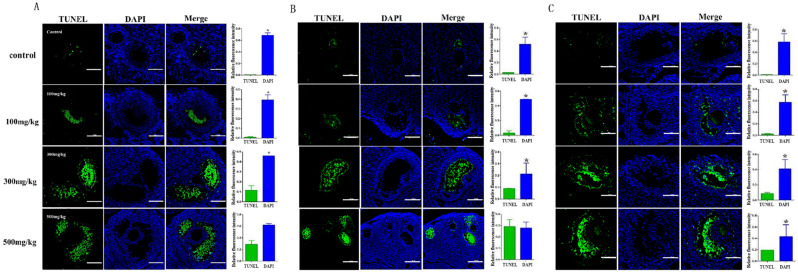
The detection of apoptosis in ovarian granulosa cells after nano-TiO_2_ gastric gavage (the white line in the figure was the scale bar, representing 100 μm). (**A**). TUNEL detection results of ovarian tissue after continuous infusion for 10 days. (**B**). Apoptotic cell-staining results of ovarian tissue at 30 days after infusion. (**C**). The detection of apoptosis in ovarian tissue at 60 days after infusion. * indicates a significant difference compared with other groups (*p* < 0.05, *n* = 5).

The expression of apoptotic cells in ovarian tissues was analyzed using the TUNEL method. At 0 days, compared with the control group, the number of apoptotic granulosa cells in the experimental group significantly increased, and this increase was further enhanced with the increase in the concentration of nano-TiO_2_ infusion (*p* < 0.05) (Figure 8A). Compared with the control group, the number of apoptotic cells increased at 30 days (Figure 8B) and 60 days (Figure 8C), and granulosa cell apoptosis continued to occur over time.

**Figure 9:**
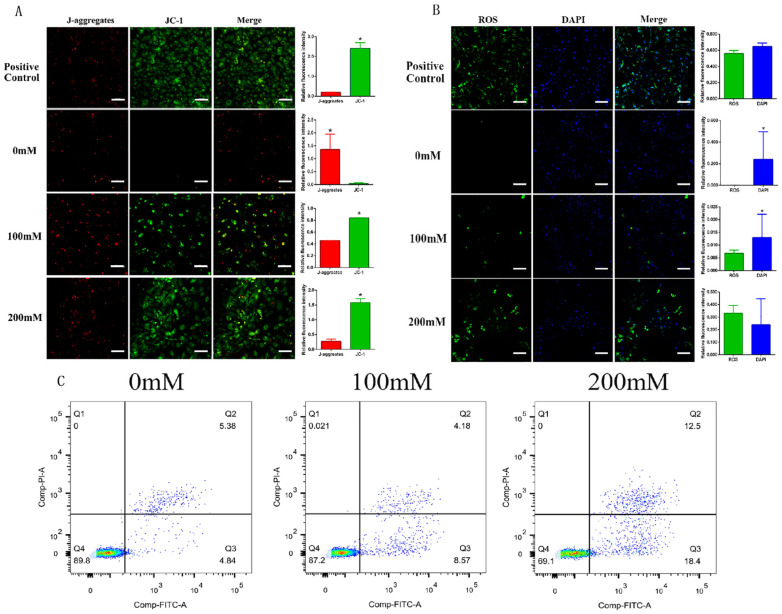
The detection of mitochondrial membrane potential and reactive oxygen species in KK1 cells after different periods of nano-TiO_2_ infusion (the scale bar is 100 μm). (**A**). The staining results of mitochondrial membrane potential after treatment with different concentrations of nano-TiO_2_ on KK1 cells. (**B**). The staining results of mitochondrial reactive oxygen species after treatment with different concentrations of nano-TiO_2_ on KK1 cells. * indicates a significant difference compared with other groups (*p* < 0.05, *n* = 5). (**C**). Flow cytometry detection results after nano-TiO_2_ intervention on KK1 cells: Q1 is the PI positive area; Q2 is the PI-FITC double-positive area; Q3 is the FITC positive area; and Q4 is the PI-FITC double-negative area.

Under normal cell growth conditions, JC-1 exists in the form of aggregates (J-aggregates). When cells undergo apoptosis, the mitochondrial membrane potential decreases, and JC-1 then changes from aggregates (red) to JC-1 monomers (green). After treating KK1 cells with nano-TiO_2_ for 24 h and detecting the mitochondrial membrane potential, the results showed that the higher the concentration of nano-TiO_2_, the more JC-1 monomers expressing green fluorescent protein, and the more significant the decrease in mitochondrial membrane potential (*p* < 0.05). Nano-TiO_2_ triggered changes in the mitochondrial membrane potential of granulosa cells and cell apoptosis (Figure 9A).

In the results of mitochondrial reactive oxygen species detection, the positive control group showed a wide distribution of ROS, high fluorescence intensity, high intracellular reactive oxygen content, and a large number of apoptotic cells. The trend of intracellular reactive oxygen content was consistent with the results of mitochondrial membrane potential detection. High concentrations of nano-TiO_2_ triggered an intracellular oxidative stress response, causing a decrease in mitochondrial membrane potential and ultimately leading to cell apoptosis (Figure 9B).

The results of the flow cytometry analysis showed that there were no PI single-positive cells. As the concentration of nano-TiO_2_ increased, the number of FITC single-positive cells gradually increased, and the number of PI-FITC double-positive cells also increased simultaneously. That is to say, nano-TiO_2_ promoted the apoptosis of granulosa cells (Figure 9C).

## 3. Discussion

The reproductive toxicity damage caused by the widespread industrial application of nano-TiO_2_ has gradually attracted attention. It has been reported that nano-TiO_2_ can inhibit the follicular development and oocyte maturation of rats [24,25], affecting development, reproduction, and movement, and its toxic effects are closely related to its physicochemical properties [26]. Our research results indicate that the infusion of nano-TiO_2_ can cause damage to the ovarian function of mice. This is consistent with the research results of Wang et al. [12,27,28,29], in which 20–50 nm titanium dioxide nanoparticles were detected to have significantly accumulated in ovarian tissue after oral exposure, and the damage to the ovarian tissue was positively correlated with the exposure dose. Due to factors such as the surface charge and lipophilicity of cells, nano-TiO_2_ particles mainly accumulate in oocytes and granulosa cells [30].

Our research results indicate that after infusion of different concentrations of nano-TiO_2_, the growth and development changes in female mice manifested as a significant down-regulation of the weight, ovarian index, littering rate, and estrogen and progesterone values in female mice; even 30 days and 60 days after the cessation of infusion, none of the above indicators showed obvious recovery signs. The potential mechanism of weight loss may be due to the accumulation of nano-TiO_2_ in the body, leading to the destruction of the intestinal barrier, inducing chronic inflammation, affecting metabolism through the hypothalamic–pituitary–adrenal axis, and causing a decrease in mitochondrial membrane potential of ovarian cells and ATP production [31]. The decline in ovarian index and birth rate is closely related to the accumulation of nano-TiO_2_ in the body, which can cause abnormal ovarian development, inhibition of steroid synthesis, and oxidative stress damage [32]. The test results of estrogen and progesterone confirm the conclusion that nano-TiO_2_ can cause reproductive endocrine disorders in female mice [33]. The research results we have presented above prove that nano-TiO_2_ damages reproductive function through the cascade reaction of oxidative stress–inflammation–endocrine disruption. The dose-dependent characteristic indicates that we need to further establish a risk assessment model based on cumulative exposure on the basis of the current research, deeply study the exposure risk of nano-TiO_2_ in food additives and daily chemical products, and explore the intervention of related antioxidants.

As our histological observation results indicate, the vacuolation of the damaged ovaries was severe and presented a dose-dependent pattern. This discovery can be explained by the fact that nanoparticles can induce excessive production of ROS, leading to lipid peroxidation and damage to the mitochondrial membrane, thereby causing cell vacuolation and even necrosis, which is consistent with the results of Liu et al. [34,35]. The explanation for our results of the reduction in follicle numbers at all levels and the phenomenon of follicle closure is that nano-TiO_2_ activates the *Bax*/*Bcl-2* pathway and caspase-3, inducing the apoptosis of granulosa cells and thereby destroying the follicular structure [36]. At the same time, nano-TiO_2_ may also excessively activate the autophagy pathway (resulting in changes such as the upregulation of LC3-II/Beclin-1), leading to premature follicle closure [37].

Consistent with the hypothesis that nano-TiO_2_ may induce programmed cell death through the mitochondrial apoptosis pathway [38], we found that in the immunohistochemical detection results, the expression of apoptotic execution proteins caspase-3 increased as the concentration of nano-TiO_2_ rose. This was the evidence that nanoparticles may induce apoptosis of ovarian cells by upregulating the Fas/FasL pathway [39]. The downregulation of the mRNA levels of specific genes in oocytes and granulosa cells was due to apoptosis, which directly reduced the overall mRNA level of the tissue, and the nano-TiO_2_-induced ROS were able to inhibit the activity of transcription factors (such as *Foxl2*) or initiate methylation in the promoter region, leading to gene silencing [40]. The specific gene-expression levels of antioxidant enzymes in decreased mice indicated that the infusion of nano-TiO_2_ caused oxidative stress responses in mice, thereby seriously affecting the normal function of the ovaries. Taking into account both the apoptotic signal expression in immunohistochemistry and the downregulation of the mRNA levels of specific factors in oocytes and granulosa cells, the association of these two results reflect the “double blow” effect of nano-TiO_2_ on the ovaries.

Our study is the first to confirm the spatial association between apoptotic signals and the functional impairment of granulosa cells at the localization level, suggesting that granulosa cells may be the primary target of nanoparticle toxicity [30]. The downregulation of granulosa cell-specific protein expression marks the beginning of ovarian dysfunction. This study found that nano-TiO_2_ exposure significantly downregulated the expression of *Foxl2* and *Inhibinα* in granulosa cells, which is consistent with the conclusion drawn by Zhou et al. [41]. Additionally, our results indicate that the apoptosis of granulosa cells is closely related to the oxidative stress response produced within the cells [42]. When granulosa cells were cultured, if the culture medium lacked the FSH factor, ROS could be detected within the cells after 4 h, and some of the granulosa cells that produced ROS had already begun to undergo apoptosis, indicating that ROS largely led to the apoptosis of granulosa cells [19]. We demonstrated that short-term (10 days) continuous exposure to nano-TiO_2_ had a certain impact on the number of follicles at all levels in mice, and the apoptotic cells were accurately identified as granulosa cells. We also proposed the hypothesis that oxidative stress responses may affect the ovarian function of mice. At the same time, the research results proved that the expression levels of antioxidant enzyme-specific genes did indeed decrease in a dose-dependent manner with the exposure to nano-TiO_2_ [23].

We used a TUNEL assay to locate the apoptotic cells, which further confirmed the hypothesis that nano-TiO_2_ promotes granulosa cell apoptosis and thereby affects follicular development. This study observed a decrease in mitochondrial membrane potential and the activation of caspase-3 in granulosa cells, suggesting that the mitochondrial-dependent apoptotic pathway was activated. This result may be due to the ROS-induced opening of the mitochondrial membrane permeability transition pore through an imbalance in *Bax*/*Bcl-2*, releasing cytochrome C and activating the caspase-9/3 cascade reaction [43] or an endoplasmic reticulum stress response and DNA oxidative damage. The secretion of maturation factors such as *Gdf9* and *Bmp15* by granulosa cells decreases, resulting in decreased oocyte quality [44], oocyte support deficiency, fibrosis, and severe functional impairment. Granulosa cell apoptosis not only directly leads to follicular closure but can also amplify ovarian damage through paracrine signals, releasing inflammatory factors such as TNF-α and IL-6, which activate the NF-κB pathway and form a pro-inflammatory-pro-apoptotic vicious cycle [45].

This study, compared to a study on the effects of low-concentration (100 mg/kg/day) nano-TiO_2_ on embryos [14], focused more on the short-term and medium–long-term (30 days, 60 days) effects of different concentrations of nano-TiO_2_ on the ovaries of mice. By using the medium–long-term culture method, we investigated the accumulation of nano-TiO_2_ in the body, which significantly affected the ovarian function and index of mice. For the first time, we used the in vitro cell-culture system to explore the effect of nano-TiO_2_ on granulosa cells, and found that nano-TiO_2_ could significantly downregulate the expression of antioxidant enzyme genes by inducing mitochondrial oxidative stress responses in mouse ovarian granulosa cells, thereby affecting follicle development [27]. Oxidative stress is the core driving factor of granulosa cell apoptosis. Nano-TiO_2_, due to its high specific surface area and photocatalytic activity, can produce ROS inside the cells, breaking through the antioxidant defense system.

During the research process, we systematically revealed the molecular mechanism by which nano-TiO_2_ damages ovarian function through the oxidative stress–particle-induced apoptosis axis, providing an important theoretical basis for the assessment of the reproductive toxicity of nanomaterials. Most existing studies adopt acute high-dose exposure models (50–300 mg/kg), while the chronic effects of low-dose long-term exposure (less than 1 mg/kg) related to the environment still need to be evaluated. In the future, we can combine single-cell sequencing, metabolomics, and other technologies to deeply analyze the toxicity pathways and promote the formulation of safety standards for nanoparticles. The research results provide key evidence for analyzing the “structural-functional dual damage” mode of nanoparticle reproductive toxicity, and are of great significance for formulating strategies to protect female reproductive health.

## 4. Materials and Methods

### 4.1. Preparation and Infusion of Nano-TiO_2_

The suspension was prepared with nano-TiO_2_ particles (formula weight: 99.87, purity: 99.8%, particle size: 20–30 nm) (T818936, Macklin, Shanghai, China) and 0.9% normal saline (Chenye, Kunming, China) at the required drug concentration, and the suspension was ultrasonic-treated for 30 min.

Sixty 5-week-old female ICR mice of similar body weight (purchased from Beijing Vitogen Life Sciences Co., Ltd. (Beijing, China), license number: SCXK (Beijing) 2021-0006) were selected.

Sixty ICR female mice were randomly divided into four groups, with 15 mice in each group. After recording the mice’s weights, ear tags were attached to mark the mice. The mice were maintained in an SPF-grade animal facility, with the temperature kept between 20 °C and 24 °C. They were kept on a natural circadian cycle and given free choice of food and water.

The amount of nano-TiO_2_ administered to each mouse in each group was calculated at 100 mg/kg/d, 300 mg/kg/d, and 500 mg/kg/d. The heads and mouths of the mice were immobilized, and gastric gavage was conducted in accordance with the assigned groups using a gavage needle to administer varying concentrations of nano-TiO_2_. Mice in the estrous cycle were selected as the research subjects for the control group and only administered 0.9% normal saline, with continuous administration for 10 days. The volume of each administration was 0.2 mL. After continuous infusion for 10 days, an interval of 24 h was included, with the date from then on recorded as day 0.

### 4.2. Cell Culture

The mouse granulosa cell line used in this study, namely the KK1 cell line, was donated by Professor Cui Sheng from the State Key Laboratory of Agricultural Biotechnology at China Agricultural University. The KK1 cells were seeded at a density of 1 × 10^6^ cells per well in 60 mm dishes. When the cells reached a confluence of 70%, the original culture medium was completely removed and replaced with sterilized nano-TiO_2_ solution and new cell-culture medium in the dishes. The dishes were then placed in the incubator for 24 h to complete the intervention. According to the residual concentration range of human exposure to nano-TiO_2_, which is 10 mm–200 mm, the experiment was set up with three groups: a blank control group, a group with a nano-TiO_2_ concentration of 0 mm, and two experimental groups with nano-TiO_2_ concentrations of 100 mm and 200 mm, respectively.

### 4.3. Hormone Testing in Mice After Nano-TiO_2_ Infusion

The mice were euthanized using the carbon-dioxide asphyxiation method. Blood was quickly collected from the apex of the heart, and the plasma was separated. The supernatant was obtained. The estradiol and progesterone contents in the mouse blood were detected using the Elisa method with the estradiol determination kit (Northern Biotechnology, Tianjin, China) and the progesterone determination kit (Northern Biotechnology, Tianjin, China). The absorbance values (OD values) were measured using an enzyme reader. The wavelength of the enzyme reader (MuLTISKAN GO, Thermo Fisher, Waltham, MA, USA) was set at 450 nm. A standard curve was plotted based on the OD values of the standard substances and the hormone concentrations, and the hormone contents were obtained.

### 4.4. Detection of Ovarian Indices and Birth Rates in Mice

The body weight of the mice was recorded before and after the infusion of nano-TiO_2_, according to the groups. After the infusion, the mice were euthanized at different times using the carbon-dioxide asphyxiation method; the mice were weighed, their ovarian tissues were extracted, the wet weight was recorded, and the ovarian index of the mice was calculated (ovarian index = ovarian weight/body weight). At the same time, in accordance with their groups, the female mice that received the nano-TiO_2_ infusion for 10 days were paired with healthy male mice with normal reproductive ability at a 2:1 ratio. Then, the number of offspring were counted for three consecutive months and a significant difference analysis was conducted.

### 4.5. Hematoxylin–Eosin Staining of Mouse Ovaries and Follicle Count

After removing the ovarian tissues of each group of mice, they were quickly immersed in 4% paraformaldehyde (PL0059, Biosharp, Baisha Li Autonomous County, China) for overnight fixation. Then, paraffin sections were prepared. After drying the sections, they underwent gradient dewaxing and hydration. After staining with hematoxylin (C0105S, Beyotime, Shanghai, China) for 5 min and eosin (C0105S, Beyotime, Shanghai, China) for 30 s, they were fully dehydrated, sealed, and subjected to HE staining for microscopic observation (LEICA, Witzla, Germany). Every 2 sections were counted once, and the number of follicles was counted at different stages of infusion.

### 4.6. Immunohistochemical Detection

The paraffin sections were deparaffinized to dehydration and then subjected to antigen retrieval in citrate buffer. Endogenous peroxidase (ZLI-9310, ZSGB-BIO, Beijing, China) underwent a reaction at 37 °C for 1 h. The sections were incubated with 10% goat serum (SP084, Solarbio, Beijing, China) in the dark for 25 min. PCNA antibody (dilution ratio of 1:200, 10205-2-AP, Proteintech, Wuhan, China), caspase-3 antibody (dilution ratio of 1:1000, 25128-1-AP, Proteintech, Wuhan, China) were added and incubated at 4 °C overnight. The next day, after the sections were re-warmed, they were incubated with the secondary antibody (SA00004-2, Proteintech, Wuhan, China) at 37 °C for 1 h. The color was developed using DAB (ZLI-9018, ZSGB-BIO, Beijing, China), and then the sections were re-stained and sealed. Observations and photos were taken under a microscope (LEICA, Witzla, Germany).

### 4.7. Apoptosis Detection of Ovarian Granulosa Cells

Detection was carried out using the TUNEL apoptosis detection kit (40306ES20, Yeasen Shagnhai, China). Firstly, tissue paraffin sections were prepared and underwent dewaxing and hydration processes. A total of 100 μL of proteinase K working solution was dropped onto each sample and incubated at room temperature for 20 min for permeabilization. The positive control group was treated with DNA enzyme I. The sample was incubated in a 37 °C water bath in the dark for 1 h with TdT. The cell nuclei were stained, sealed, and observed under a fluorescence microscope.

### 4.8. RT-qPCR

Extract total RNA from ovarian tissues and perform reverse transcription: Take the ovarian tissues from each group of mice, extract the total RNA from the ovarian tissues using the Trizol (9109, TaKaRA, Shiga Prefecture, Japan) method, and reverse-transcribe it into cDNA (RR047Q, TaKaRa, Shiga Prefecture, Japan). Quantify the RNA concentration to 1 μg/μL. The primers for the target genes were synthesized by Shanghai Sangon Biotech (Shanghai, China) (Table 1).

RT-qPCR: A total of 40 cycles were carried out using the 10 μL setting of TB Green premix Ex Taq II (RR820A, TaKaRA, Shiga Prefecture, Japan), starting at 95 °C for 5 s, then 60 °C for 30 s, then 72 °C for 30 s. The dissolution curve, showing a single peak, indicates the good specificity of the PCR reaction. Three replicates were set for all experiments, and the average value was taken. After the reaction was completed, statistical analysis was conducted on the data using *GAPDH* as the reference gene. The relative expression level of the gene was calculated by the calculation method of 2^−ΔΔCt^, and the expression level of the blank control gene was set to 1.00.

### 4.9. Mitochondrial Membrane Potential Detection (JC-1)

The detection was carried out using the mitochondrial membrane potential detection kit (JC-1) (C2006, Beyotime, China). Firstly, cell slides were prepared on a 6-well plate coated with gelatin, with the KKI cell density set at 1 × 10^5^. The cells were treated with different concentrations of nano-TiO_2_ according to the groups, and a positive control group was set up. The cells were stained with the JC-1 working solution and incubated for a period of time. After thorough washing of the cells, the reaction was terminated by adding culture medium, and the slides were sealed for observation under a laser confocal microscope and recorded.

### 4.10. Mitochondrial ROS Detection

The cells were spread onto the slides and then subjected to nano-TiO_2_ intervention. Positive controls were set up according to the steps necessary for employing the mitochondrial reactive oxygen species detection kit (S0033, Beyotime, Shanghai, China), probes were loaded in situ, incubated, and thoroughly washed, the cell nuclei were re-stained, and the slides were sealed, observed, and recorded under a laser confocal microscope.

### 4.11. Flow Cytometry (FCM) of FITC-PI Apoptotic Cell Detection

According to the set-up procedure of the Annexin V-FITC Apoptosis Detection Kit (CA1020, Solarbio, Beijing, China), we suspended the cells, adjusted the cell density to 1 × 10^6^ cells/mL, added 100 μL of cell suspension to each flow tube, clearly labeled them, then added 5 μL of Annexin V-FITC to the tubes. After incubating at room temperature in the dark for 10 min, we added 5 μL of PI solution, placed the flow tube on ice, incubated it at room temperature in the dark for 5 min, detected the apoptosis level using a flow cytometer, and performed data analysis using FlowJo software (V10.0).

### 4.12. Data Statistical Analysis

The data were processed and analyzed using SPSS 22.0. The experimental data were expressed as mean ± standard deviation ($\bar{X}$ ± S). According to the experimental design, the comparison and analysis of data between groups were conducted using the SNK method of one-way analysis of variance (ANOVA) for multiple comparisons. *p* < 0.05 indicated a statistically significant difference.

## 5. Conclusions

The accumulation of nano-TiO_2_ in the body leads to the induction of cell apoptosis in granulosa cells, as well as the inhibition of the expression of specific genes in oocytes and granulosa cells, resulting in damage to ovarian structures and functional decline. This toxic mechanism may stem from the “double blow” effect mediated by oxidative stress, directly disrupting cellular homeostasis and simultaneously interfering with the transcription of reproductive-related genes, highlighting the potential risks of nanomaterial exposure to female reproductive health. Through the research on mouse models, we have provided reliable laboratory research basis for establishing the safe usage range of nanomaterials for humans and formulating industry standards for nanomaterials. Given the limited intervention time of the nano-TiO_2_ in this study, we need to conduct further verification and improve other experiments such as antioxidant rescue, in order to provide ideas for finding intervention measures to prevent and control the harm caused by nanomaterials to the human body.

## Figures and Tables

**Table 1:** The sequence of the primers used for PCR.

Target Gene	Primer Sequence	Product Size (bp)	Annealing Temperature (°C)
*GAPDH*	F: AGGTCGGTGTGAACGGATTTG R: TGTAGACCATGTAGTTGAGGTCA	123	58
*Oct4*	F: GATGCTGTGAGCCAAGGCAAG R: GGCTCCTGATCAACAGCATCAC	145	58
*Bmp15*	F: TCCTTGCTGACGACCCTACAT R: TACCTCAGGGGATAGCCTTGG	100	55
*Gdf9*	F:TCTTAGTAGCCTTAGCTCTCAGG R: TGTCAGTCCCATCTACAGGCA	116	55
*Foxl2*	F:ACAACACCGGAGAAACCAGAC R: CGTAGAACGGGAACTTGGCTA	145	55
*Foxo3*	F: CTGGGGGAACCTGTCCTATG R: TCATTCTGAACGCGCATGAAG	210	55
*Inhibinα*	F: GCACAGGACCTCTGAACCAG R: GGGATGGCCGGAATACATAAG	101	60
*Zp3*	F: ATGGCGTCAAGCTATTTCCTC R: CGTGCCAAAAAGGTCTCTACT	186	55
*Bax*	F: TGAAGACAGGGGCCTTTTTG R: AATTCGCCGGAGACACTCG	140	60
*Bcl-2*	F:ATGCCTTTGTGGAACTATATGGC R: GGTATGCACCCAGAGTGATGC	120	55
*Sod2*	F: ATGGTGGGGGACATATT R: GAACCTTGGACTCCCACAGA	167	55
*Cat*	F: CCTCGTTCAGGATGTGGTTT R: TCTGGTGATATCGTGGGTGA	130	57
*Gpx*	F: GTCCACCGTGTATGCCTTCT R: TCTGCAGATCGTTCATCTCG	152	57

## Data Availability

The authors confirm that all data generated or analyzed during this study are included in this published article (and its Supplementary Information Files).

## Supplementary Materials

The following supporting information can be downloaded at: https://www.mdpi.com/article/10.3390/ijms26146981/s1.
