# Supplementary material for: Nano-Titanium Dioxide Induces Ovarian Function Damage in Mice by Mediating Granulosa Cell Apoptosis
Source: Int J Mol Sci. 2025 Jul 20;26(14):6981. doi: 10.3390/ijms26146981 (PMC12295327; doi:10.3390/ijms26146981)
Supplement: Supplementary file 1 [file ijms-26-06981-s001.zip › ijms-3697341 -English-Editing-Certificate-97141.pdf]

We certify that the following article

Nano-titanium dioxide induces varian function damage in mice by mediating granulosa cell apoptosis

Jie Chen, Shengbo Zhang, Changbao Wu, Yaxuan Zhang, Jingyu Ren, Xiaoxiao You, Yanfeng Dai *

has undergone English language editing by MDPI. The text has been checked for correct use of grammar and common te[illegible] terms, and edited to a level suitable for reporting research in a scholarly journal.

MDPI uses experienced, native English speaking editors. Full details of the editing service can be found at ► https://www.mdpi.com/authors/english.

MDPI

Basel, Switzerland
July 2025

AUTHORservices

english-97141
